# Weight Gain in a Sample of Patients Affected by Overweight/Obesity with and without a Psychiatric Diagnosis during the Covid-19 Lockdown

**DOI:** 10.3390/nu12113525

**Published:** 2020-11-16

**Authors:** Serena Marchitelli, Cristina Mazza, Andrea Lenzi, Eleonora Ricci, Lucio Gnessi, Paolo Roma

**Affiliations:** 1 Department of Experimental Medicine, Sapienza University of Rome, 00185 Rome, Italy; serena.marchitelli@uniroma1.it (S.M.); andrea.lenzi@uniroma1.it (A.L.); lucio.gnessi@uniroma1.it (L.G.); 2 Department of Neuroscience, Imaging and Clinical Sciences, “G. d’Annunzio” University of Chieti-Pescara, 66100 Chieti, Italy; eleonoraricci25@gmail.com; 3 Department of Human Neuroscience, Sapienza University of Rome, 00185 Rome, Italy; paolo.roma@uniroma1.it

**Keywords:** COVID-19, overweight, obesity, weight gain, psychological distress

## Abstract

The present study aimed at identifying psychological and psychosocial variables that might predict weight gain during the COVID-19 lockdown in patients affected by overweight/obesity with and without a psychiatric diagnosis. An online survey was administered between 25 April and 10 May 2020, to investigate participants’ changes in dietary habits during the lockdown period. 110 participants were recruited and allocated to two groups, 63 patients had no psychiatric diagnosis; there were 47 patients with psychiatric diagnosis. ANOVA analyses compared the groups with respect to psychological distress levels, risk perception, social support, emotion regulation, and eating behaviors. For each group, a binary logistic regression analysis was conducted, including the factors that were found to significantly differ between groups. Weight gain during lockdown was reported by 31 of the participants affected by overweight/obesity without a psychiatric diagnosis and by 31 patients with a psychiatric diagnosis. Weight gain predictors were stress and low depression for patients without a psychiatric diagnosis and binge eating behaviors for patients with a psychiatric diagnosis. Of patients without a psychiatric diagnosis, 60% reported much more frequent night eating episodes. The risk of night eating syndrome in persons affected by overweight/obesity with no psychiatric diagnosis should be further investigated to inform the development of tailored medical, psychological, and psychosocial interventions.

## 1. Introduction

In early 2020, the 2019 coronavirus disease (COVID-19) pandemic began to spread across Italy. To limit the spread of the virus, on 9 March, the Italian government introduced several strict containment measures that drove the nation into a complete lockdown. Movement outside residences was forbidden throughout the country, except for critical tasks related to work, food, and health. Furthermore, physical distance between persons was enforced. Prior to COVID-19, the most recent global epidemic requiring containment measures was SARS (2002–2003), which involved the lockdown of some cities in East Asia. In Italy, however, the COVID-19 lockdown measures were unprecedented.

In the past months, several global surveys have been conducted to determine the impact of the COVID-19 containment measures—particularly self-isolation and social distancing—on behavior, well-being, and daily life [1,2,3]. Such surveys have shown that the strict lockdown measures have a severe negative psychological impact on patients with psychiatric disorders [4]. Moreover, fear of the disease, the general lockdown situation, high levels of uncertainty regarding the future, and financial insecurity have increased stress, anxiety, and depression, even in the nonpsychiatric population [1,5,6,7]. In addition to these global surveys, studies have also examined the effect of the lockdown on several specific subgroups (e.g., patients with psychiatric disorders), in order to better understand the full impact of the pandemic. One such subgroup is that of patients affected by morbid obesity, who are considered at high risk of contracting COVID-19. In fact, research suggests that patients affected by morbid obesity with COVID-19 are twice as likely to die than those who are not affected by overweight. Moreover, this excess mortality is both direct—resulting from the viral infection itself—and indirect—resulting from the virus’s effect on other medical conditions, including increased obesity [8]. 

Obesity is a major public health problem that has been increasing in prevalence for the past 30 years. According to the World Health Organization, in 2017, more than 1.9 billion people were affected by overweight and 600 million by obesity [9]. In Western Europe, in particular, obesity affects 10–20% of the population. Obesity is associated with reduced economic and social opportunities and quality of life, and it is a determinant of several intermediate risk factors associated with increased mortality and lower life expectancy. The most severe form of obesity, morbid obesity, is a multifactorial and complex metabolic disease that is defined by a body mass index (BMI) of more than 40 kg/m^2^, or more than 35 kg/m^2^ with comorbidities. As a result of various lifestyle changes, the prevalence of morbid obesity has been rising across the world, particularly in America and Europe, and it has become one of the most life-threatening medical conditions. In particular, general obesity is a risk factor for cardiovascular disease, type 2 diabetes, orthopedic problems, and some oncologic diseases; it is also associated with psychosocial comorbidities, underachievement in school and/or work, unstable or poor relationships, lower self-esteem, excessive focus on body image or body shape, and poor quality of life [10]. Furthermore, it is often comorbid with several psychiatric disorders, including major depressive disorder and dysthymic disorder, anxiety disorders (social phobia or generalized anxiety disorder), eating disorders (binge eating disorder, but also pervasively disordered eating behavior with alternating restricting and binge eating), personality disorders (histrionic, borderline, and schizotypal), and substance abuse [10,11,12]. As recently shown by some surveys on COVID-19 lockdown and eating disorders, individuals with eating disorders are at significant risk of negative impacts of the pandemic [13]. Indeed, it has been reported that patients with bulimia nervosa demonstrated exacerbated binge eating during the lockdown period [14].

Following the findings of the more recent surveys on the effect of COVID-19 lockdown among this specific population, the main aim of the present study was to investigate the impact of psychological and psychosocial variables on weight gain in patients affected by overweight/obesity not only with but also without a psychiatric diagnosis, over the COVID-19 lockdown. Additionally, we explored changes in the sample’s dietary habits over the lockdown.

## 2. Materials and Methods

### 2.1. Procedures

An online survey was created on the basis of the literature, and data were collected from 25 April to 10 May 2020, during the COVID-19 lockdown in Italy that started on 9 March and ended on 18 May 2020. The questionnaire was administered on an online survey platform, which participants accessed via a designated link. Participants voluntarily responded to the anonymous survey and indicated their informed consent prior to providing feedback. According to the European General Data Protection Regulation (GPDR; 2016/679 of The European Parliament and of The Council of 27 April 2016), in the informed consent form it was specified that the patient’s name would never appear anywhere, nor would the answers provided by the patient be attributable to the patient. After the data were collected, an anonymous identification code was associated, and any other data/information that could lead to the patient was deleted. All procedures were clearly explained, and participants could interrupt or quit the survey at any point without explaining their reasons for doing so.

The study was approved by the Institutional Board of the Department of Human Neuroscience, Faculty of Medicine and Dentistry, “Sapienza” University of Rome (IRB-2020-6), in conformity with the principles embodied in the Declaration of Helsinki.

### 2.2. Participants

All participants were inpatients of C.A.S.C.O. (“Centro di Alta Specializzazione per la Cura dell’Obesità e delle malattie correlate”)—a medical center specialized in treating obesity—at the University Hospital “Policlinico Umberto I” of Sapienza University, Rome. At C.A.S.C.O., all patients receiving day hospital treatment undergo endocrinological, cardiological, metabolic, internistic, nutritional, and psychological screenings prior to being “prescribed” a nutritional diet, bariatric surgery (i.e., gastric bypass, sleeve gastrectomy), or endoluminal procedures. The screening of psychiatric diagnosis was conducted through the routine psychiatric assessment, which includes a general, medical, and psychiatric history and the mental status examination of the patient.

Patients who had accessed C.A.S.C.O. between March 2019 and February 2020 were invited via telephone to participate in the research (*n* = 242). Of the 242 patients who were approached, 182 patients agreed to participate. Patients who had undergone bariatric surgery or endoluminal procedures (*n* = 43) were excluded, due to the direct effect of such procedures on eating behaviors. The remaining 139 patients had not undergone bariatric surgery or endoluminal procedures over the prior year and agreed to participate. All 139 patients were following a diet under C.A.S.C.O. supervision. Twenty-nine patients (out of the 139) did not complete the online survey. Thus, the final sample was composed of 110 participants and allocated to two groups, 63 patients without a psychiatric diagnosis and 47 patients with psychiatric diagnosis. Specifically, the psychiatrics comorbidities of our sample included the following: eating disorders (e.g., binge eating disorder, night eating syndrome, bulimia nervosa), mood disorders (e.g., major depressive disorder, bipolar disorder), anxiety disorders (e.g., generalized anxiety disorder, panic disorder), substance use disorders, and psychotic disorders. All patients were aged 18 years or older and living in Italy.

### 2.3. Survey Measures

In addition to a questionnaire that was designed to collect participants’ demographic data (i.e., age, education, marital status, occupation, citizenship, and region of residence), specific experiences of the COVID-19 lockdown (e.g., *Who are you spending lockdown period with?*; *Have you had loved ones (e.g., family members, friends) infected with COVID-19*?; *Have you ever had psychotherapy?; Have you ever taken psychopharmacological medications?),* and changes in dietary habits (e.g., *During the lockdown, have the episodes of compulsive binge eating increased?; During the lockdown, has the food intake at meals increased?; During the lockdown, has the food intake between meals increased?; During the lockdown, has the intake of snacks and junk food increased?; During the lockdown, have the night eating episodes increased?*), the following measures were employed.

### 2.4. The General Health Questionnaire-12

The General Health Questionnaire-12 (GHQ-12) [15] was adopted to detect psychiatric disorders of a nonpsychotic nature. The GHQ-12 is a self-administered questionnaire comprising 12 items (example item: *Since the COVID-19 emergency started, have you felt constantly under pressure?*) that are measured on a 4-point Likert scale ranging from 1 (less than usual) to 4 (much more than usual). The GHQ-12 has been shown to be a reliable instrument, as indicated by a Cronbach’s alpha of 0.84 [16,17,18]. In our sample, the GHQ-12 obtained high reliability, with Cronbach’s alpha of 0.86.

### 2.5. The Depression, Anxiety, and Stress Scale—21 Items

The Depression, Anxiety, and Stress Scale—21 Items (DASS-21) [19] was used to assess participants’ mental health. The DASS-21 is a set of three self-report scales designed to measure the emotional states of depression (example item: *In the last 7 days, I couldn’t seem to experience any positive feeling at all*), anxiety (example item: *In the last 7 days, I was worried about situations in which I might panic and make a fool of myself*), and stress (example item: *In the last 7 days, I tended to over-react to situations*). All subscales are rated on a 4-point Likert scale ranging from 0 (never) to 3 (almost always). The DASS-21 obtained high reliabilities in the Italian validation study, with Cronbach’s alphas of 0.74, 0.82, and 0.85 for the Anxiety, Depression, and Stress subscales, respectively; Cronbach’s alpha for the total scales was 0.90 [19]. In our sample, Cronbach’s alphas were 0.92, 0.86, and 0.89 for the Stress, Anxiety, and Depression subscales, respectively.

### 2.6. Risk Perception

Risk perception was assessed on the basis of two variables, perceived severity and perceived likelihood, using items adapted from Cho and Lee [20] and Liao et al. [21]. Perceived severity was assessed through four items (example item: “*If I got COVID-19, it would be severe*”), while perceived likelihood was assessed through two items (example item: “*How likely is it that you will get COVID-19 in this period*?”). All six items were assessed on a 5-point Likert scale ranging from 1 (not likely at all) to 5 (certain). The Cronbach’s alpha from Cho and Lee’s [20] study was 0.90 for the Korean sample and 0.78 for the US sample. In our sample, Cronbach’s alpha was 0.78.

### 2.7. The Social Connections (SC) Subscale

Adapted from the Parents’ Assessment of Protective Factors (PAPF) [22], the SC subscale was administered to assess subjects’ social support. The SC subscale is composed of nine items (example item: “*I have someone who will help me get through tough times*”) that are rated on a 5-point Likert scale ranging from 0 (*this is not at all like me or what I believe*) to 4 (*this is very much like me or what I believe*). The Social Connections subscale has shown high reliability, as indicated by a Cronbach’s alpha of 0.93 [22]. In our sample, Cronbach’s alpha was 0.95, showing high reliability.

### 2.8. The Binge Eating Scale

The Binge Eating Scale (BES) [23,24] was used to assess the behavioral manifestations of binge eating (e.g., eating quickly and overeating) and the feelings/cognitions associated with binge eating (e.g., feeling guilty after binge eating) [23]. The BES is a 16-item self-report measure. For each item, respondents select one of three or four response options (example item with three response options: *I don’t feel guilty at all, nor do I hate myself after eating too much; Sometimes I feel guilty or hate myself after eating too much; I almost always experience a strong sense of guilt or hate myself after eating too much*), coded 0–2 or 0–3, respectively. Scores are summed to produce a total score in the range of 0–46. Three levels of binge eating severity are defined: none-to-minimal (<17 total score), moderate (18–26), and severe (>27) [25]. In the original study [23], the BES was found to have good validity and internal consistency (α = 0.85). In our sample, Cronbach’s alpha was 0.90, showing high reliability.

### 2.9. The Difficulties in Emotion Regulation Scale

The Difficulties in Emotional Regulation Scale (DERS) [26,27] was employed to assess multiple aspects of emotion dysregulation. The Italian DERS is a 36-item self-report questionnaire, which yields a total score as well as six subscale scores relating to non-acceptance of emotional responses (example item: *When I’m upset, I become angry with myself for feeling that way*), difficulties engaging in goal-directed behavior (example item: *When I’m upset, I have difficulty getting work done*), lack of trust in one’s own emotional regulation skills (example item: *When I’m upset, it takes me a long time to feel better*), difficulties in controlling behavior (example item: *When I’m upset, I become out of control*), difficulties in emotion recognition (example item: *I am confused about how I feel*), and reduced emotional awareness (example item: *When I’m upset, I acknowledge my emotions*). All subscales are rated on a 5-point Likert scale ranging from 1 (never) to 5 (almost always). In the Italian validation study, the DERS demonstrated high reliability, with Cronbach’s alpha of 0.90 [27]. In our sample, Cronbach’s alphas for the subscales ranged from 0.74 (difficulties in emotion recognition subscale) to 0.91 (non-acceptance of emotional responses subscale). Cronbach’s alpha for the total scale was 0.92.

### 2.10. Statistical Analysis

ANOVA analyses were conducted to compare the two groups (i.e., with and without a psychiatric diagnosis) with respect to psychological distress levels (i.e., GHQ-12; DASS-21 stress, depression and anxiety scales), risk perception, binge eating behaviors (i.e., BES scale), social support and emotion regulation difficulties (i.e., DERS scale). We also inspected the effect sizes of the score differences between groups. Values of 0.02, 0.13, and 0.26 were considered indicative of small, medium, and large effects, respectively [28]. It has been calculated that a sample size of 110, divided into two groups, is sufficiently large to achieve, in ANOVA analysis, at least a statistical power (1-β) = 0.95, given a significance level α = 0.05 and an effect size 0.40 [29]. Following this, for each group, a binary logistic regression analysis was run; measures found to significantly differ between groups were entered as predictors and weight gain (no weight gain vs. weight gain) was entered as the dichotomous dependent variable. The Hosmer–Lemeshow test was used to assess goodness of fit for logistic models, which evaluates whether predicted probabilities agree with observed ones; it should be nonsignificant for an accurate predictive model [30]. The SPSS-25 statistical package (SPSS inc., Chicago, IL, USA) was used for all analyses.

## 3. Results

Participants were allocated to one of two groups according to the presence or absence of a psychiatric diagnosis. The 63 patients without a psychiatric diagnosis included 42 females (66.7%) and 21 males (33.3%), aged 18–75 years (M = 47.24, SD = 14.3), with an average BMI of 40.19 kg/m^2^ (SD = 6.8, range: 27–60). Of these, 3 (4.8%) were patients with overweight, 8 (12.7%) with obesity class I, 26 (41.3%) with obesity class II, and 26 (41.3%) with obesity class III.

The 47 patients with a psychiatric diagnosis included 36 females (76.6%) and 11 males (23.4%), aged 18–74 years (*M* = 46.38, *SD* = 14.5), with an average BMI of 39.88 kg/m^2^ (*SD* = 6.8, range: 28–55). Of these, 3 (6.4%) were patients with overweight, 6 (12.8%) with obesity class I, 12 (25.5%) with obesity class II, and 26 (55.3%) with obesity class III. Psychiatric diagnoses included the following (even in comorbidity): mood disorders (e.g., major depressive disorder, bipolar disorder), eating disorders (e.g., binge eating disorder, night eating syndrome, bulimia nervosa), anxiety disorders (e.g., generalized anxiety disorder, panic disorder), substance use disorders, and psychotic disorders. Of patients with overweight/obesity and a psychiatric disorder, 80% had a Binge Eating Disorder (BED) as main diagnosis or in comorbidity.

The groups did not differ in age (*p* = 0.758), gender (*p* = 0.257), BMI (*p* = 0.806), educational level (*p* = 0.662), marital status (*p* = 0.576), occupation (*p* = 0.583), use of psychiatric drugs (*p* = 0.080), or obesity classes (*p* = 0.364); however, they did differ in their use of psychotherapy (*p* = 0.006), as was expected.

Weight gain over the lockdown was reported by the half of the participants affected by overweight/obesity without a psychiatric diagnosis (*n* = 31 out of 63) and by 31 (out of 47) patients with a psychiatric diagnosis.

More descriptive statistics are presented in Table 1. Information about changes in dietary habits over the lockdown is shown in Table 2.

The results revealed statistically significant differences for the DASS-21 Depression, DASS-21 Stress, and BES scores, with a small effect size for the DASS-21 subscales and a medium effect size for the BES differences. In detail, the group of patients affected by overweight/obesity and with a psychiatric diagnosis demonstrating significantly higher values than the group of patients affected by overweight/obesity without a psychiatric diagnosis (Table 3).

**Figure 1:**
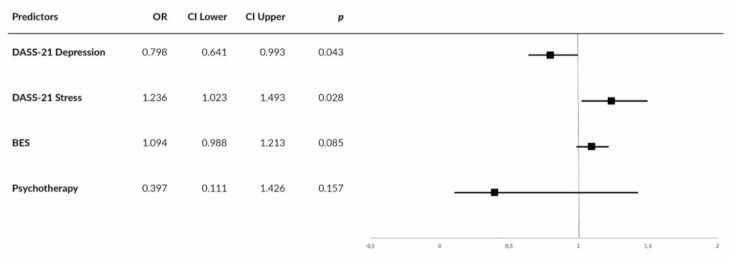
Forest plot showing center-specific ORs and 95% CIs for DASS-21 Depression, DASS-21 Stress, Binge Eating Scale (BES), and use of psychotherapy in patients affected by overweight/obesity without a psychiatric diagnosis.

A binary logistic regression analysis was performed using the enter method. Weight gain (no weight gain vs. weight gain) was set as the dichotomous dependent variable, while the DASS-21 Stress and DASS-21 Depression scores, the BES total score, and the use of psychotherapy (past or current) were used as covariates. The inserted variables were those that showed a statistical difference in the previous multivariate ANOVAs and *x*^2^ comparing the two groups. The Wald test was used to evaluate the contribution of each individual predictor to the model. A predictor was entered into the regression equation when the probability (*p*) reached 0.05. For the group without a psychiatric disorder, overall prediction success was 69.8% (78.1% for no weight gain and 61.3% for weight gain). The prediction model showed goodness of fit to the observed data (χ^2^(4) = 11.379, *p* = 0.023, Hosmer–Lemeshow test of goodness of fit: χ^2^(8) = 8.253, *p* = 0.409). Nagelkerke’s R of 0.220 indicated a relationship—albeit weak—between prediction and grouping. The final prediction model comprised the DASS-21 Stress and Depression scores; the BES total score was excluded (Figure 1).

**Figure 2:**
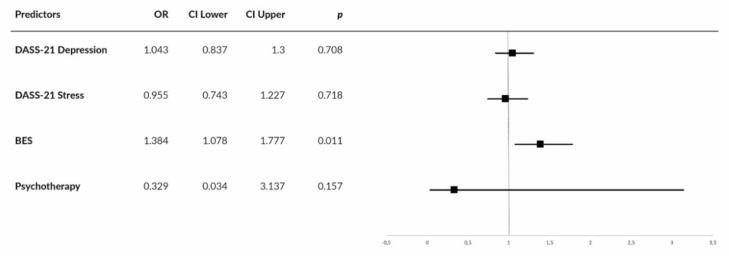
Forest plot showing center-specific ORs and 95% CIs for DASS-21 Depression, DASS-21 Stress, Binge Eating Scale (BES), and use of psychotherapy in patients affected by overweight/obesity with a psychiatric diagnosis.

For the group with a psychiatric diagnosis, overall prediction success was 83% (75% for no weight gain and 87.1% for weight gain). The prediction model showed goodness of fit to the observed data (χ^2^(4) = 25.802, *p* < 0.001, Hosmer–Lemeshow test of goodness of fit: χ^2^(7) = 7.518, *p* = 0.377). Nagelkerke’s R of 0.585 indicated a moderate relationship between prediction and grouping. The final prediction model comprised the BES total score, only; both DASS-21 scores were excluded (Figure 2).

## 4. Discussion

Among the surveyed patients affected by overweight/obesity, 50% of those without a psychiatric diagnosis and 66% of those with a psychiatric diagnosis reported weight gain during the COVID-19 lockdown. The present study sought to investigate the impact of psychological and psychosocial variables on weight gain in patients affected by overweight/obesity, both with and without a psychiatric diagnosis, over the COVID-19 lockdown. Concurrently, it explored these patients’ changes dietary habits.

The results showed higher levels of stress and depression in patients affected by overweight/obesity with a psychiatric diagnosis compared with those with no psychiatric diagnosis. However, despite this significant difference between groups, levels of psychological distress (relating to depression, stress, and anxiety) in the psychiatric patients affected by overweight/obesity were lower than the averages reported for the clinical population [19,31,32]. Furthermore, increased psychological distress during the COVID-19 lockdown did not seem to predict weight gain for these patients; instead, they were more likely to gain weight as a result of binge eating behaviors, which preceded the lockdown situation and were frequently a symptom of their psychiatric diagnosis.

For patients affected by overweight/obesity without a psychiatric diagnosis, psychological stress was the best predictor of weight gain during the COVID-19 lockdown. Specifically, a rise in stress during the lockdown—which has also been found in the general Italian population [6,7,33,34,35,36,37,38]—may have triggered an increase in night eating episodes. Surprisingly, approximately 60% of the overweight and obese patients with no prior diagnosis of night eating syndrome [39] reported much more frequent night eating episodes during the lockdown. This finding suggests that the lockdown situation may be increasing the prevalence of nutrition and eating disorders (particularly night eating syndrome) in persons with obesity, including those with no prior history of binge eating.

The relationship of mood disorders with obesity has been the subject of many studies. Depression has been shown to be frequently associated with obesity [40], and it often presents with comorbid binge eating; these conditions are both known to lead to poorer results in weight loss programs [41]. On the other hand, low levels of depression, as those shown by our results, could be interpreted as a sign of hypomanic activation, like explained by Amianto et al. [42] in their study with patients with a subthreshold binge eating disorder. Further studies should investigate the implications of these findings in order to understand how the lockdown conditions may be contributing to the development of new eating habits, and to establish whether these new habits are likely to be longstanding or permanent.

The present results should be interpreted with caution, due to some limitations of the study. First, the cross-sectional study design, implemented during the first phases of the COVID-19 outbreak in Italy, prevented us from drawing causal inferences (i.e., we were unable to assess respondents’ psychological functioning prior to the pandemic). Further, data were collected via a web-based survey that relied on voluntary sampling and self-reported data; thus, the data may have been distorted by selection or social desirability biases. Social desirability could have interfered especially in reporting weight gain, given that patients were following a nutritional diet under C.A.S.C.O. supervision. This social desirability bias may have been also the reason why some patients withdrew from the survey. Finally, the final sample was small (*n* = 110) and mainly composed of females who lived in the center of Italy; greater analysis of patients who undergo different treatment programs throughout the Italian peninsula may have provided additional insight and a more comprehensive picture of the psychological situation of this specific population.

Despite these limitations, the results of the study represent an important starting point. In fact, with a view to increasing the spread of telemedicine, the use of web technologies can be useful to monitor the psychophysical state of patients with a more constant and gradual frequency, weighing less on the health system, already burdened from the COVID-19 emergency. Furthermore, since 60% of patients without a psychiatric diagnosis reported much more frequent night eating episodes, and consequently are at greater risk to develop a night eating syndrome, our results support the importance to make a dimensional psychological/psychiatric diagnosis, which may lead to greater attention being paid to this specific subclinical population.

## 5. Conclusions

The present findings suggest that there is a need for further investigation into changes in eating behavior during the COVID-19 lockdown amongst patients affected by overweight/obesity with and without a psychiatric diagnosis. In particular, nonpsychiatric with overweight/obesity persons’ greater risk for developing night eating syndrome points to the need for further research into the precise lockdown conditions (e.g., disrupted circadian rhythms) that might explain an increase in binge episodes at night. Other variables related to the lockdown (e.g., social isolation) [43] or not related to the COVID-19 outbreak (e.g., be waiting for bariatric surgery or endoluminal procedures) should also be investigated to determine their contribution—if any—to the observed change in eating behaviors. Further, future research should seek to compare the present study data with that collected using other methods (e.g., semistructured interviews, qualitative approaches). Finally, given the recent resurgence of COVID-19, it would be useful to conduct a further study to identify any additional changes in eating behavior of patients affected by overweight/obesity at this time, in order to guide the development of tailored medical, and psychological, psychosocial interventions.

## Figures and Tables

**Table 1:** Descriptive statistics of the sample of patients affected by overweight/obesity with and without a Psychiatric Diagnosis.

Characteristic	Group	without a Psychiatric Diagnosis (*n* = 63) *n* (%)	with a Psychiatric Diagnosis (*n* = 47) *n* (%)
**Citizenship**	Italian	60 (95.2)	45 (95.7)
	Foreign	3 (4.8)	2 (4.3)
**Region of residence**	Center	61 (96.8)	43 (91.5)
	South/islands	2 (3.2)	4 (8.5)
**Education**	Middle school diploma	16 (25.4)	11 (23.4)
	High school diploma	24 (38.1)	23 (48.9)
	Graduate	14 (22.2)	7 (14.9)
	Postgraduate	9 (14.3)	6 (12.8)
**Marital status**	Unmarried	18 (28.6)	14 (29.8)
	Married	30 (47.6)	22 (46.8)
	Cohabitant	5 (7.9)	5 (10.6)
	Divorced	1 (1.6)	3 (6.4)
	Separated	5 (7.9)	1 (2.1)
	Widower	4 (6.3)	2 (4.3)
**Occupation**	Employee	34 (54)	19 (40.4)
	Freelancer	7 (11.1)	6 (12.8)
	Unemployed	14 (22.2)	13 (27.7)
	Student	2 (3.2)	4 (8.5)
	Retired	6 (9.5)	5 (10.6)
**Spending social distancing period with**	Alone	8 (12.7)	7 (14.9)
	Family	36 (57.1)	29 (61.7)
	Partner	13 (20.6)	11 (23.4)
	Roommate(s)	3 (4.8)	
	Child(ren)	2 (3.2)	
	Other(s)	1 (1.6)	
**Use of psychotherapy**	Never	42 (66.7)	19 (40.4)
	Previously/currently	21 (33.3)	28 (59.6)
**Use of psychopharmacology**	Never	53 (84.1)	33 (70.2)
	Previously/currently	10 (15.9)	14 (29.8)
**Infected acquaintances or loved ones**	No	57 (90.5)	41 (87.2)
	Yes	6 (9.5)	6 (12.8)
**Deaths among infected**	No	60 (95.2)	46 (97.9)
**acquaintances or loved ones**	Yes	3 (4.8)	1 (2.1)

**Table 2:** Changes in dietary habits during the lockdown.

		Patients without a Psychiatric Diagnosis (*n* = 63) *n* (%)	Patients with a Psychiatric Diagnosis (*n* = 47) *n* (%)	*p*
**Increased compulsive binge eating**	I have never practiced compulsive binge eating	9 (14.3%)	5 (10.6%)	0.276
	Not at all	29 (46%)	17 (36.2%)	
	Rarely	16 (25.4%)	11 (23.4%)	
	Often	7 (11.1%)	13 (27.7%)	
	Very often	2 (3.2%)	1 (2.1%)	
**Increased food intake at meals**	Not at all	25 (39.7%)	14 (29.8%)	0.553
	Rarely	28 (44.4%)	22 (46.8%)	
	Often	8 (12.7%)	10 (21.3%)	
	Very often	2 (3.2%)	1 (2.1%)	
**Increased food intake between meals**	I have never eaten between meals	7 (11.1%)	2 (4.3%)	0.297
	Less frequent	11 (17.5%)	7 (14.9%)	
	Equal	22 (34.9%)	12 (25.5%)	
	More frequent	20 (31.7%)	21 (44.7%)	
	Much more frequent	3 (4.8%)	5 (10.6%)	
**Increased intake of snacks and junk food**	I have never eaten snacks or junk food	13 (20.6%)	7 (14.9%)	0.373
	Less frequent	15 (23.8%)	10 (21.3%)	
	Equal	18 (28.6%)	11 (23.4%)	
	More frequent	17 (27%)	17 (36.2%)	
	Much more frequent	0	2 (4.3%)	
**Increased night eating episodes**	I have never got up at night to eat	12 (19%)	5 (10.6%)	0.085
	Less frequent	11 (17.5%)	17 (36.2%)	
	Equal	2 (3.2%)	0	
	More frequent	0	1 (2.1%)	
	Much more frequent	38 (60.3%)	24 (51.1%)	

**Table 3:** Mean differences between patients affected by overweight/obesity with and without a psychiatric diagnosis.

Scale	Cronbach’s Alpha	without a Psychiatric Diagnosis (*n* = 63) *M* (*SD*)	with a Psychiatric Diagnosis (*n* = 47) *M* (*SD*)	*p*	*parn^2^*
GHQ-12 total score	0.86	17.83 (5.7)	19.49 (6.4)	0.154	0.019
DASS-21 Depression	0.89	4.16 (4.6)	6.4 (5.3)	0.019	0.050
DASS-21 Anxiety	0.86	2.81 (3.5)	4.06 (4.7)	0.113	0.023
DASS-21 Stress	0.92	5.67 (4.9)	7.98 (6)	0.028	0.044
Risk Perception	0.78	19.48 (4.2)	19.96 (4.4)	0.565	0.003
BES	0.90	7.89 (6.9)	14.4 (11.2)	<0.001	0.116
none-to-minimal level		55 (87.3%)	32 (68.1%)		
moderate level		7 (11.1%)	8 (17%)		
severe level		1 (1.6%)	7 (14.9%)		
SC subscale	0.95	2.8 (1.2)	2.67 (1.1)	0.564	0.003
DERS total score	0.92	71.92 (20.9)	78.32 (23.8)	0.138	0.020
Non-acceptance of emotional responses	0.91	10.51 (5.1)	12.70 (6.5)	0.051	0.035
Difficulties engaging in goal-directed behavior	0.89	10.56 (4.9)	12.49 (5.8)	0.062	0.032
Lack of trust in one’s own emotional regulation skills	0.84	15.89 (6.5)	17.30 (7.4)	0.293	0.010
Difficulties in controlling behavior	0.83	9.73 (3.8)	10.43 (5)	0.410	0.006
Difficulties in emotion recognition	0.74	10.41 (4.3)	9.85 (4.1)	0.490	0.004
Reduced emotional awareness	0.80	7.19 (3.6)	6.85 (3.1)	0.606	0.002

Note. GHQ-12: General Health Questionnaire; DASS-21: Depression Anxiety Stress Scale—21 Items; BES: Binge Eating Scale; SC Subscale: Social Connections Subscale; DERS: Difficulties in Emotion Regulation Scale; *parn^2^*: partial eta squared—effect size.
